# Next-generation synthetic trials in hematology with generative artificial intelligence

**DOI:** 10.1038/s41375-026-03116-9

**Published:** 2026-08-19

**Authors:** Jan-Niklas Eckardt, Martin Bornhäuser, Jan Moritz Middeke

**Affiliations:** 1 Department of Internal Medicine I, University Hospital Carl Gustav Carus, TUD Dresden University of Technology, Dresden, Germany; 2 Else Kröner Fresenius Center for Digital Health, TUD Dresden University of Technology, Dresden, Germany; 3 Mildred Scheel Early Career Center, National Center for Tumor Diseases (NCT/UCC) Dresden, Faculty of Medicine and University Hospital Carl Gustav Carus, TUD Dresden University of Technology, Dresden, Germany; 4 German Cancer Consortium (DKTK), Partner Site Dresden, and German Cancer Research Center (DKFZ), Heidelberg, Germany; 5 National Center for Tumor Diseases (NCT), NCT//UCC Dresden, a partnership between DKFZ, Faculty of Medicine and University Hospital Carl Gustav Carus, TUD Dresden University of Technology, and Helmholtz-Zentrum Dresden-Rossendorf (HZDR), Dresden, Germany

**Keywords:** Haematological cancer, Clinical trials

## A need for new trial designs in the era of targeted therapies

**Fig. 1:**
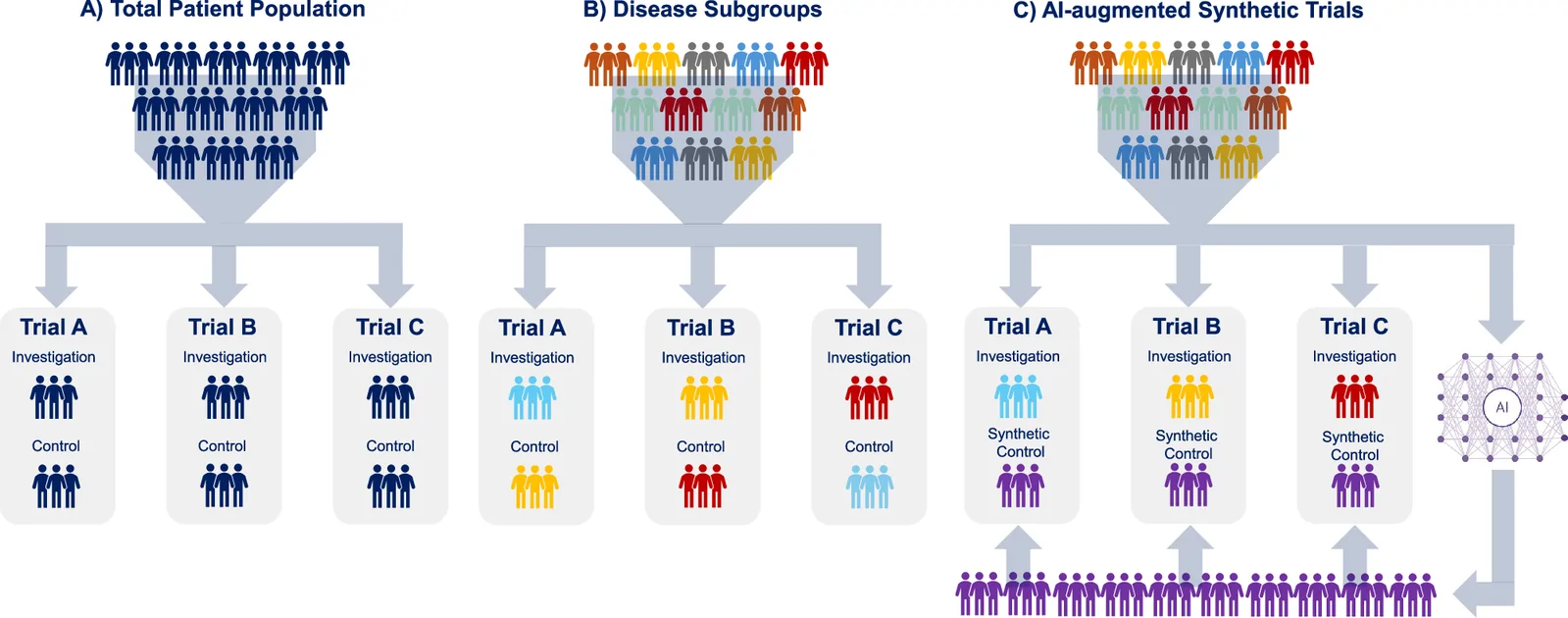
Three generations of control arm design. In the past, trial inclusion and exclusion criteria often did not consider molecularly-defined subgroups, essentially rendering the entire patient population of a certain hematological entity as potential candidates for trial inclusion (**A**). With the advent of modern sequencing techniques came the fragmentation of cancer entities into subgroups according to diverging pathomechanisms, which could increasingly be exploited via targeted therapies. This, however, enables trials to “cannibalize” each other, as patients recruited into the standard-of-care (often chemotherapy-based) control arm of one trial, are lost from the investigational arm of another trial they could have been a suitable subgroup candidate for (**B**). Synthetic patient data generated by artificial intelligence could enable the generation of large representative synthetic patient cohorts drawn from previous clinical trials and real-world registries. By reflecting the standard of care, these synthetic cohorts could substitute for, or replace, control arms in precision hematology trials, enabling eligible subgroup patients to be enrolled in investigational arms (**C**). Created with Microsoft PowerPoint.

Developing new cancer drugs requires deep pockets, iron patience, and a level of frustration tolerance that matches the Greek mythos of Sisyphos: The budget needed to bring a single cancer drug to market is estimated to exceed a billion US dollars, spent over almost a decade in development time, and more often than not the undertaking still ends in failure [1–4]. One of the top reasons why cancer trials frequently falter is recruitment failure [5]. The situation will only worsen given the ongoing fragmentation of cancer entities into molecularly-defined subgroups addressed by targeted agents in the era of precision oncology. When multiple trials compete for the same shrinking pool of potentially eligible patients, the demand for individual control arms becomes a problem rather than a perk: a patient recruited into the control arm of one trial is a patient lost from the investigational arm of another. Trials begin to “cannibalize” each other, contributing to slower accrual, thereby increasing the likelihood of trial failure. Compounding this challenge, the therapeutic standard frequently changes during the course of a trial due to the approval of new treatments or changes in risk classification. Collectively, this slows the generation of evidence across the portfolio of trials, ultimately delaying or even denying patients access to potentially effective therapies (Fig. 1). So, if a standard-of-care control group for every trial remains a practical and regulatory necessity, can we create one instead of recruiting one?

## Generative artificial intelligence enables the creation of synthetic patient cohorts

Generative artificial intelligence (AI) can now create synthetic data indistinguishable from real data, including text, images, audio, video, and tabular data. The underlying principle is a statistical mimicry of feature distributions in real data that are approximated by neural networks to create synthetic samples following the same distributions without being exact copies of their original training data. In cancer research, this includes the generation of medical images (e.g., histopathology or cytomorphology), text data from electronic health records, or tabular data including laboratory parameters, genetics, and outcomes. Importantly, this concept of synthetic data differs from digital twins: The latter constitute a digital replica of an individual patient to dynamically model trajectories given certain interventions. Synthetic cohort data reproduce feature distributions across a cohort, thereby capturing statistical properties and inter-feature relationships rather than producing a mirror image of a single patient. Arguably, synthetic patient data may therefore also provide a safeguard to patient privacy. Synthetic samples can hardly be traced back to the original patient data they have been created with (more on this later), thereby reducing barriers in health data access and sharing. While the concept of synthetic data generation has long existed, advances in AI architectures, software frameworks, and computing power have enabled widespread training and implementation of generative models. Frequently used model architectures include generative adversarial networks (GANs), variational autoencoders, diffusion models, and transformers.

In hematology, AI increasingly supports physicians in diagnosis and therapeutic decision-making [6]. We and others have demonstrated that synthetic data faithfully reproduce disease properties observed in real patient cohorts, can be used for translational research, and may potentially substitute or replace control cohorts in clinical trials. For instance, we trained GAN models to generate synthetic bone marrow smears that experts failed to distinguish from real samples and used these synthetic images to train highly accurate image classifiers for leukemia detection in microscopy [7]. Further, we generated synthetic patients trained on 1,606 acute myeloid leukemia (AML) patients from previous clinical trials of the German Study Alliance Leukemia, consisting of multimodal tabular data including clinical, laboratory, and genetic features. The resulting synthetic cohorts recapitulated real patient properties, disease biology, and survival dynamics [8]. More recently, we used synthetic AML patients to retrospectively replace the control cohort of the phase 2 trial SORAML [9] (which evaluated the addition of sorafenib to standard induction therapy), effectively reproducing original trial outcomes when comparing the original intervention to the synthetic control arm [10]. Similarly, Piciocchi et al. [11] generated synthetic AML patients based on a cohort of 500 patients from the GIMEMA AML1310 trial, reporting survival dynamics matching original trial outcomes. In myelodysplastic neoplasms (MDS), D’Amico et al. [12] created a large synthetic cohort of patients to investigate prognostically relevant features, which, in their analysis overlapped with the IPSS-M features [13], suggesting the IPSS-M could have also been discovered in a synthetically augmented cohort.

## Pitfalls and the path forward for synthetic data in clinical trials

**Fig. 2:**
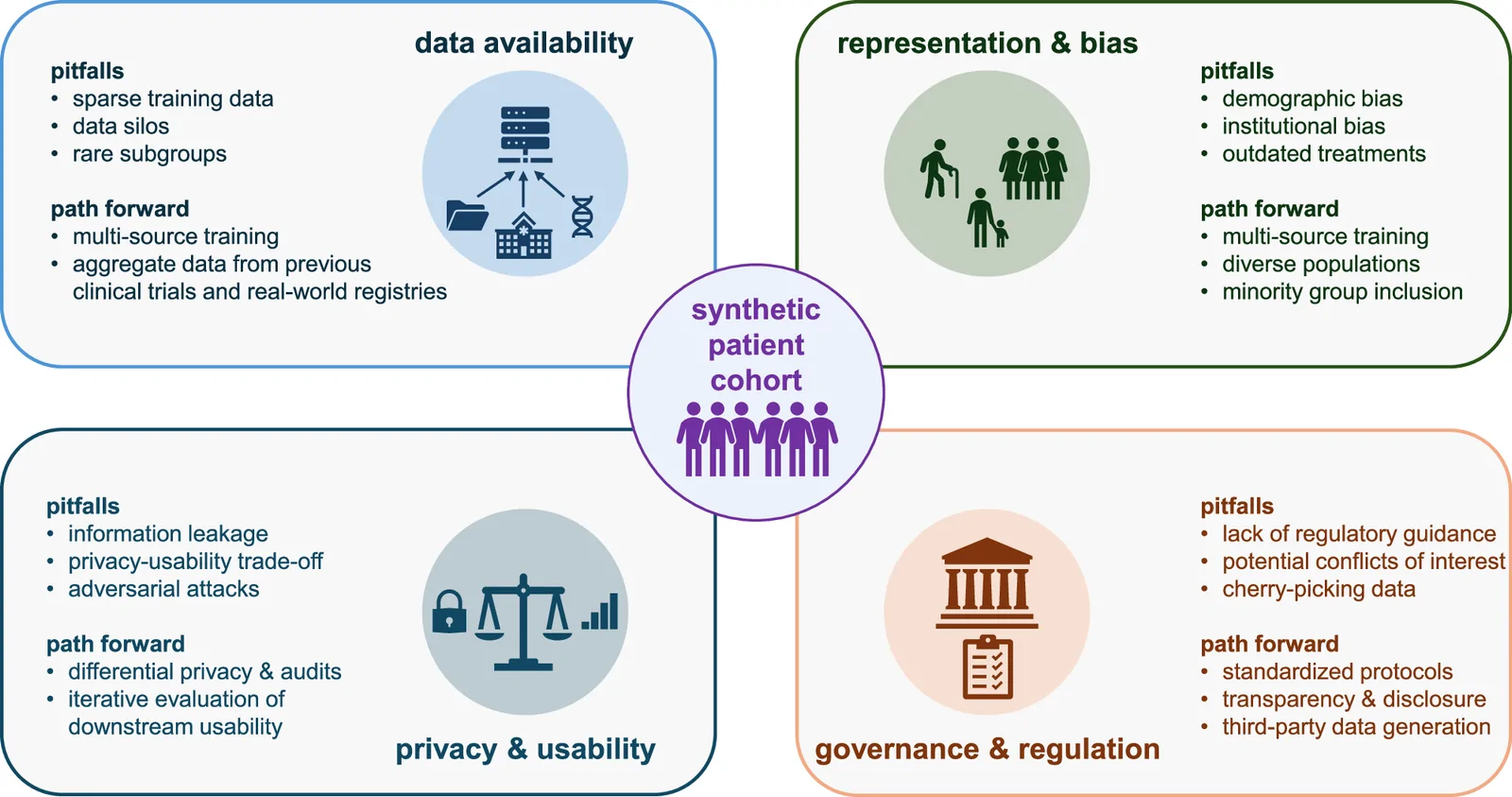
Pitfalls and the path forward for synthetic data in clinical trials. Off-the-shelf synthetic data are no silver bullet: Synthetic data could amplify existing biases in medicine, lead to further underrepresentation of minority groups, leak sensitive patient information, and allow for adversarial behavior. Hence, following best practices is essential, including multi-source training, deliberate inclusion of minority populations, privacy audits and safeguards, and transparent disclosure of training data properties and model settings. In the setting of clinical trials, commercial or academic conflicts of interest may require synthetic data generation by third parties to mitigate cherry-picking and uphold regulatory standards. Created with Microsoft PowerPoint.

Despite their potential, synthetic patient data are no panacea, requiring clinicians and researchers to be aware of potential pitfalls and adhere to best practices for implementation (Fig. 2). First, data generation itself is locked in a conundrum: Synthetic data may enable privacy-compliant health data access and sharing as well as facilitate novel trial designs, yet to train a generative model, one needs a large, diverse, and representative training sample so that the model can infer accurate feature distributions and capture intricate relationships. Generative models cannot make up useful data from scratch. If one desires to generate synthetic data of a specific group of patients, yet one has no access to a sufficiently sized training cohort in the first place, no generative model will be able to create said group of patients from thin air. Hence, the first step is defining the relevant task to be addressed with synthetic patients and then identifying a group of patients that can actually be synthetically created. For clinical trials, this may best apply to patients receiving standard of care since plenty of patient records for training exist across healthcare systems, previous trials, and registries.

Second, generative models mirror feature distributions of their training data. Hence, they may also carry over or even amplify implicit biases, including local patient demographics, institutional preferences in treatment selection, or specific properties of subgroups if they are overrepresented. This is further complicated by inadvertently modeling confounding covariates or neglecting unknown or unknowable covariates modifying statistical properties. Training data should therefore come from multiple sources - ideally not only relying on patient data from previously conducted clinical trials but also include real-world registries [14, 15] - to ensure adequate sample size, representativeness, and generalizability [16]. All variables should be assessed for interference or redundancies prior to model training. The inclusion of minority groups is of particular importance, as otherwise generative models will not contribute to solving the equity problem in medicine but will quietly intensify it.

Third, by modeling the underlying distribution of features and creating synthetic patient samples that fit this distribution, synthetic data are not exact replicas of the training data patients. Yet, privacy preservation is not guaranteed by default. Sensitive information can still be exposed either through unintended model behavior or adversarial manipulation, such as membership inference or model inversion attacks [17, 18]. Safeguarding patients’ privacy should not be an afterthought, but privacy audits should rather guide the data generation process from inception. Crucially, one must account for a privacy-usability tradeoff: The more synthetic data differ from original data, the more privacy-compliant they become, yet the less useful they are for downstream tasks. In the absence of universally accepted thresholds for adequate privacy preservation, potential information leakage and downstream usability must be iteratively assessed throughout generation. Differential privacy budgets, real-to-synthetic distance metrics, and design safeguards combatting adversarial attacks help mitigate the risk of privacy breaches [19, 20].

Lastly, synthetic data are in a regulatory limbo. Regulatory agencies increasingly acknowledge the need for alternative control cohorts in settings where placebo control is not feasible, or recruitment is slowed by small or inaccessible patient populations, especially in rare cancers or molecular subgroups [21, 22]. However, currently no regulatory framework exists for synthetically controlled trials. Regulators will have to define appropriate quality measures, similar to existing frameworks such as the Food and Drug Administration’s “Good Machine Learning Practice for Medical Device Development”[23]. These should include transparency requirements on training cohort properties, potentially arising limitations and biases, disclosure of model architecture, metrics for fidelity and usability, as well as privacy preservation [24, 25]. Crucially, synthetic data generation allows a degree of customization, e.g., by generating large cohorts and selecting only those cases with the desired properties. In clinical trials, this may lead to a critical conflict of interest as entities with commercial or academic stakes in the outcome of a trial could potentially “cherry-pick” a synthetic control cohort to manufacture a desired result. Regulatory agencies should therefore provide a framework for synthetic data generation by independent third parties, with the resulting cohort withheld from investigators and sponsors until completion of intervention arm data collection.

In summary, synthetic data hold the potential to reduce barriers in data sharing and may enable novel trial designs, accelerate recruitment, reduce failure rates, and provide more patients with access to investigational therapies, particularly in the era of precision therapies in hematology. Yet, they are no silver bullet: Rigorous evaluation, quality assessment, privacy preservation, and regulatory guidance are needed before clinical implementation.
